# Associations Between Swimmers’ Dry-Land Lower- and Upper-Limb Measures and Butterfly Sprint Performance

**DOI:** 10.3390/sports13100346

**Published:** 2025-10-03

**Authors:** Maciej Hołub, Wojciech Głyk, Arkadiusz Stanula, Katja Weiss, Thomas Rosemann, Beat Knechtle

**Affiliations:** 1 Institute of Sport Sciences, Academy of Physical Education, 40-065 Katowice, Poland; m.holub@awf.katowice.pl (M.H.); w.glyk@awf.katowice.pl (W.G.); a.stanula@awf.katowice.pl (A.S.); 2 Medbase St. Gallen Am Vadianplatz, 9000 St. Gallen, Switzerland; katja@weiss.co.com (K.W.); thomas.rosemann@usz.ch (T.R.); 3 Institute of Primary Care, University of Zurich, 8091 Zurich, Switzerland

**Keywords:** swimming sports performance, butterfly sprint, lower and upper limbs’ characteristics, dolphin kick, dry-land training, vertical jump

## Abstract

The aim of the study was to determine correlations between performance of vertical jumps and dolphin kick sprints, and between the results of a dry-land butterfly arm pull test and butterfly arms-only swimming. The study recruited competitive junior male swimmers (15.9 (0.7) years, 179.3 (5.3) cm body height, 64.6 (4.3) kg body mass). On dry land, we measured jump height, lower-limb work and power, as well as peak velocity, power, and force in the butterfly arm pull test. In swimming tests, time, velocity, power, force, and work were assessed during the dolphin kick and butterfly arms-only trials. Pearson’s correlation coefficients and the coefficients of determination were calculated between measurements. The findings showed correlations between swimming velocity and power recorded during the dolphin kick test with jump height, work and power measured in the jump tests (maximum *r* = 0.90, *r*^2^ = 0,81, *p* < 0.05). The best correlations between the results of the jump tests and swim variables were determined for the CJ30s test. The butterfly arm pull test was not associated with all parameters measured by the butterfly arms-only test. Our study demonstrates that targeted dry-land training programmes using exercises like vertical jumps can enhance competitive swimmers’ performance and offer coaches an accessible means of tracking athlete progress. Moreover, such simple drills may serve as a cost-effective approach for early evaluation of strength and power potential and for preventing musculoskeletal injuries, all without requiring pool access or specialized underwater equipment. However, the small and homogeneous sample (*n* = 12, junior males only) and the absence of reliability analyses limit the generalizability of the results.

## 1. Introduction

Swimming is one of the most popular sports worldwide. A measure of its popularity is swimming events at the Olympic Games that draw larger audiences than many other events [1]. Its attractiveness, changing competition rules, new technologies, and the coaches’ increasing skills and expertise contribute to the development of competitive swimming and enable swimmers to perform at an increasingly high level [2,3]. Competitive swimming is also a strenuous sport that carries a risk of injury related to repetitive cyclic movements of the limbs performed by swimmers with high intensity [4]. Swimming coaches increasingly realise the potential of dry-land training to improve swimmers’ fitness, make them less prone to injuries, and reduce the pool training time [5].

Dry-land strength exercises included in professional training programmes have been shown to have many benefits for competitive swimmers [6,7], ranging from improved swimming performance to athletes’ better physiological and biomechanical characteristics [8]. Moreover, simple strength and endurance tests (e.g., vertical jumps) constitute a cost-effective and widely accessible means for the early assessment of strength and power potential, as well as the prevention of musculoskeletal injuries, since they allow fitness training and monitoring without the need for pool access or expensive underwater equipment. It is also essential that coaches and athletes recognize the utility of such tests—thus allowing their use as indicators of an athlete’s overall health status rather than solely as measures of sport-specific performance. It needs to be noted, however, that their effectiveness depends on how well they reflect the structure of propulsive movements in the water [9]. The vertical jump test is widely viewed as a reliable measure of explosive anaerobic power [10], and its results are considered good predictors of the performance of competitive and recreational athletes [11]. The advantages of the test appear to be very natural regarding sports involving vertical jumps, but they seem less obvious in the case of sports requiring diverse movement patterns, such as swimming. Also, compared with the number of studies reporting an association between vertical jump performance and the performance of some elements of competitive swimming, such as a dive off the block [12,13,14], studies investigating relationships between vertical jump and legs-only swimming are few. However, there were moderate to strong positive associations between the work during vertical jumps and tethered swimming forces, particularly in whole-body and legs-only swimming [15]. This research gap is surprising because most of the power of the dolphin kick comes from the knee extensors [16], which also generate the largest proportion of power during vertical jumps [17].

The butterfly stroke, which is rarely used by swimmers other than competitive athletes [18], and breaststroke are symmetric swimming styles that, even in the 1950s, were classified as the same swimming style [2]. They are similar indeed in that both belong to the most strenuous and technically demanding styles that require good coordination of movements and strong upper limbs, which have been found to account for 90% of swimming velocity in boys [19]. The strong association between swimming velocity and the strength of the upper limbs prompts an interesting question about whether the force generated by the swimmer’s arms in the water and performing swimming-like movements on dry land is similar.

Given the above, this study sought to determine if there were correlations between dry-land exercises and swimming over short distances, specifically between the performance of vertical jumps and dolphin kick sprints, and between the results of a dry-land butterfly arm pull test and butterfly arms-only swimming. The justification for including both single and continuous jump tests was strengthened by explicitly linking them to existing research gaps. This clarification highlights how our study addresses the lack of evidence on the predictive value of these dry-land measures for legs-only and butterfly-specific swimming performance. It is hypothesized that correlations exist between the investigated variables, suggesting that incorporating appropriately selected dry-land exercises into in-water training programs would enhance swimmers’ motor skills and overall fitness.

## 2. Materials and Methods

The study set out to determine: (a) relationships between jump height and lower limb work and power measured with dry-land jump tests and biomechanical variables recorded during dolphin kick tests in the water; (b) relationships between the peak values of upper limbs’ power, force, and velocity measured with a dry-land butterfly arm pull test and biomechanical variables recorded during butterfly arms-only swims. The significance of these relationships would mean that enhancing swimming training programmes with lower and upper limb exercises would benefit swimmers and that dry-land tests reliably measure and predict their performance.

### 2.1. Subjects

The study recruited 12 competitive junior male swimmers (15.9 (0.7) years, 179.3 (5.3) cm body height, 64.6 (4.3) kg body mass, 20.2 (1.2) BMI) attending a swim class at a sports school in Poland and having swimming skills qualifying them to compete in national junior events. The recruitment period was conducted on a single day, specifically on 10 January 2022. Before enrolment, they were familiarised with the study’s design, and consent forms signed by their legal guardians were collected. The protocol of the study conformed to the 1975 Declaration of Helsinki as revised in 2000 and was approved by the Bioethics Committee for Scientific Research at the Jerzy Kukuczka Academy of Physical Education in Katowice (No. 8/2018). Informed consent was obtained from the parents or legal guardians of any participant under the age of 16.

### 2.2. Procedures

The tests were preceded by a dynamic warm-up closely supervised by the investigators, because warm-up quality influences the reliability of measurements. Static stretching exercises were omitted due to their potentially adverse effect on jump performance. The participants were thoroughly briefed on the correct performance of the jumps before the tests began [20].

#### 2.2.1. The Dry-Land Tests

Dry-land tests included a squat jump (SJ), a countermovement jump (CMJ), an akimbo countermovement jump (ACMJ), 15 s of continuous jumps (CJ15s), and 30 s of continuous jumps (SJ30s). Measurements were taken using the OptoJump Next device (Microgate, Bolzano, Italy). The tests were administered at 10 min intervals to prevent participants’ increasing fatigue from biasing their results [21]. The OptoJump Next device was connected by a cable to a laptop computer with dedicated software (version 1.12.21), which displayed the actual height of jumps (the CMJ, ACMJ, and SJ tests) and their average height (the CJ15s and CJ30s tests) in centimetres (cm). The software also computed average lower limb power during the CJ15s and CJ30s tests and presented it in W∙kg^−1^. The height of single jumps was calculated using the Sayers formula:

PAPw (Watts) = 60.7 − jump height (cm) + 45.3 − body mass (kg) − 2055

Lower limb work during the jump tests was derived from the mgΔh formula, where “m” stands for body mass (kg), “g” is acceleration (m∙s^−2^), and Δh indicates the elevation of the centre of gravity (m). The CJ15s and CJ130s tests were conducted as a single test because having participants perform them separately on the same day would be too strenuous for them. The approach also ensured participants’ full commitment and helped them concentrate their strength and endurance on one measurement instead of two. Each single jump test was performed twice, and the better of 2 trials was recorded for analysis. The continuous jump test was measured separately for 15 and 30 s.

The Squad Jump (SJ) test required a subject standing with the knees and hips fully extended, feet approximately shoulder-width apart [14], to bend the knees at 90° for 2–3 s and then jump as high as he could with hands placed on the hips [22].

The Countermovement Jump (CMJ) was a vertical jump that a subject performed with an arm swing countermovement after flexing the knees to 90° [23,24]. The biomechanics of the CMJ is the most similar to that of a natural human jump, and the height and power recorded tend to be greater compared with other jumps [25]. Because the CMJ test yields reliable and reproducible results, it is a valuable source of information about muscular power [26,27]. The validity and reliability of vertical jump tests (SJ, CMJ, ACMJ) have been well established in previous research, with excellent reproducibility (ICC ≥ 0.80, CV ≤ 10%) and internal consistency (Cronbach’s α > 0.95) reported across multiple studies [28,29].

The Akimbo Countermovement Jump (ACMJ) test was performed in much the same way as the CMJ test, the only difference being that it required a subject to keep their hands on the hips [30]. As the ACMJ does not involve an arm swing, the jump heights it measures tend to be lower compared with the CMJ [31].

The CJ15s and CJ30s tests were conducted as a single test requiring a subject to perform maximal continuous vertical jumps for 30 s; the first 15 s were measured as a separate test. While jumping, a subject kept hands on the hips, the trunk as upright as possible [32], and the knees flexed at approximately 90° in the transition between the eccentric-concentric phases [33]. The two tests are more convenient to apply than the longer tests and are less expensive compared with other methods of anaerobic power assessment because they do not need sophisticated equipment to be carried out [34].

The peak values of power, force, and velocity of participants’ arms during the butterfly stroke were measured with the butterfly arm pull test. It was conducted using the Sprint 1080 device (1080 Motion, Lidingo, Sweden) [35] and a line resistance corresponding to 10% of a subject’s body mass. The measurement was taken while a subject was lying in the prone position on a bench elevated at a 30° angle, holding the exercise bar attached to the cord of the Sprint 1080 device with hands placed shoulder-width apart. On the investigator’s signal, a subject pulled the bar as far down as possible with the butterfly arm movement. Two trials separated by a 15 s rest break were allowed. The Sprint 1080 system has also been shown to be a valid and reliable device for monitoring sprint-related performance variables, with reported ICC values between 0.86 and 0.95 and CV around 1–2% [36,37].

#### 2.2.2. The Swim Tests

The 25 m dolphin kick test and the 25 m butterfly arms-only test were conducted in an Olympic-size pool (50 m long). They were preceded by individual warm-ups to ensure that the participants would perform at their best. The test protocols were designed by the investigators. Measurements were taken with the Sprint 1080 device, which recorded the swimmers’ times [s], velocity [m∙s^−1^], power [W], force [N], and work [J] for distances of 0–5, 0–10, 0–15 and 0–20 m, and calculated their mean values. The first 5 m after a push-off from the wall were omitted from the analysis.

The Sprint 1080 was mounted on top of the wall opposite to that where the participants started the test pool. The hip belt at the end of the cable protruded 40 cm from the device for easy attachment. The measurement was activated by a pull on the cord and continued until the end of the set distance. A 1 kg line resistance was set for all participants.

The 25 m dolphin kick test started with a subject standing with their back against the pool wall. On the investigator’s signal, a subject pushed off from the wall and swam as fast as he could, holding a kickboard with fully extended arms.

The 25 m butterfly arms-only test consisted of sprint swimming with a pull buoy between the thighs. The starting position and the manner of measurement were the same as during the dolphin kick test.

### 2.3. Statistical Analysis

The results of all tests are presented below as means ± standard deviations. The normality of their distributions was determined using the Shapiro–Wilk test. The descriptive statistics were calculated for swimmers’ age, height and weight, and the results of the tests. Pearson’s correlation coefficients and the coefficients of determination were calculated between the results of the SJ, CMJ, ACMJ, CJ15s, and CJ30s tests (jump height and lower limb work and power) and time, velocity, power, strength, and work obtained with the 25 m dolphin kick test, as well as between the peak values of power, force, and velocity yielded by the butterfly arm pull test and the 25 m butterfly arms-only test. The level of statistical significance was set at *p* < 0.05. The results of the tests were analysed in Statistica 13.1 software (TIBCO Software Inc., Palo Alto, CA, USA).

## 3. Results

With regard to the single jump tests, the highest values of jump height and lower limb work and power were obtained for the CMJ test (39.28 (6.74) cm, 3224.42 (568.33) W, and 249.96 (46.28) J, respectively; Table 1). Those yielded by the ACMJ test were lower by 1.53 cm (4.05%), 92.57 W (2.96%), and 9.92 J (4.13%). The lowest values, approximately 14–20% lower compared to the CMJ test, were measured in the SJ test. A comparison of the CJ15s and CJ30s tests showed that higher values were yielded by the former: 27.8 (5.35) cm, 20.03 (3.37) W, and 176.69 (37.08) J. Values recorded during the CJ30s test were lower by 2.52 cm (9.97%), 1.43 W∙kg^−1^ (7.69%), and 115.81 J (9.83%). The peak values of power, force, and velocity measured by the butterfly arm pull test (Table 2) were 659.27 (122.97) W, 144.81 (29.25) N, and 5.05 (1.37) m∙s^−1^, respectively.

As the data in Table 3 show, the fastest swimming time, 4.85 (0.56) s, was obtained for the 0–5 m distance. The times measured for the next 5 m distances were 9.93 s, 15.35 s, and 21.15 s, respectively, meaning that they were longer by 0.23 s (5.08 s), 0.34 s (5.42 s), and 0.38 s (5.80 s). The same pattern occurred for swimming velocity, which was the highest for the first distance (1.05 (0.13) m∙s^−1^ and then kept declining from distance to distance by exactly 0.03 m∙s^−1^ (1.02, 0.99, and 0.96 m∙s^−1^). The peak values of power (18.52 (2.53) W, 0.47, 0.53, and 0.64) also successively decreased by 18.05, 17.52 and 16.98 W, respectively. The peak force that was the greatest for the first, fastest distance, 15.78 (0.62) N, decreased for the next distances to 15.71 N (−0.7), 15.64 N (−0.7), and 15.58 N (−0.6). Similarly, the greatest value of work was measured for the first distance: 88.64 (6.64) J; its values for the next distances were progressively smaller, i.e., 88.20 J, 87.78 J, and 87.71 J (by 0.44, 0.42, and 0.07 J, respectively).

According to Pearson’s *r*, the height of jumps and lower limb work and power measured during the jump tests were not associated with force and work recorded in the dolphin-kick test, the peak values of power, force, and velocity recorded during the butterfly arm pull test, and all parameters measured by the butterfly arms-only test.

**Figure 1:**
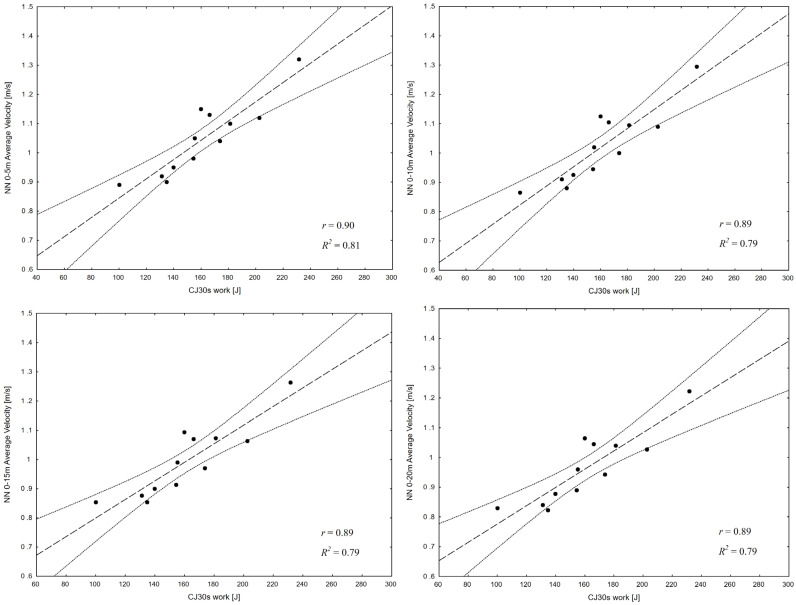
A linear relationship between lower-limb work [J] during the CJ30s test and swimming velocities measured during the dolphin kick test [m∙s^−1^] by distance (based on Pearson’s correlations and coefficients of determination) with dashed lines indicating the 95% confidence interval.

The greatest values of *r* and *R*^2^ in Table 4 are 0.90 and 0.81. Only 6 of the 180 Pearson’s correlations are not significant (*p* > 0.05). 120 parameters (i.e., all relating to time and swimming velocity) have *r* exceeding 0.71. This indicates that the studied correlations are strong, but the focus of further analysis will be on the strongest ones, with *r* above 0.8. Strong correlations occurred between lower limb work and power in the CJ30s test and swimming times and velocities measured for all distances, including the longest one (0–20 m; Figure 1). In all cases, Pearson’s *r* for work ranges from 0.88 to 0.90 (*R*^2^ = 0.76 to 0.81); for power, it is from 0.87 to 0.88 (*R*^2^ = 0.75 to 0.78).

**Figure 2:**
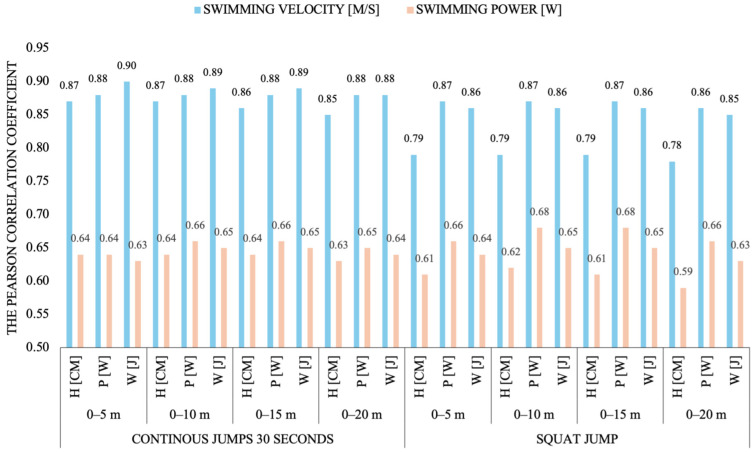
Pearson’s correlations (*r*) between average swimming velocity [m∙s^−1^] and power [W] measured during the dolphin kick test by distance and average jump height [cm], power [W] and work [J] in the CJ30s and SJ tests.

Equally high Pearson’s correlations, almost identical to those obtained for the CJ30s test, were calculated for the CJ15s parameters and swimming time and velocity, particularly for lower limb work and power (*r* = 0.81 to 0.87; *R*^2^ = 0.65 to 0.76). The height of jumps was less correlated with swimming performance than work and power; however, the height of the continuous jumps was more strongly correlated with it than the height of the single jumps (Figure 2). With regard to the single jump test, very strong correlations were calculated between lower limb work and power measured by the SJ and ACMJ tests and swimming times and velocities for each distance *r* = 0.79 to 0.87; *R*^2^ = 0.63 to 0.76); a similarly high correlation (*r* > 0.8) was not obtained for jump height and swimming velocity. The CMJ test proved to be the least correlated with swimming performance because the correlations between its parameters and the swimming variables never exceeded 0.8.

## 4. Discussion

Main findings of this study were focused on examining the relationship between the results of dry-land fitness tests and the performance in a butterfly arms-only test and a dolphin kick test, both conducted in the pool. Based on the identified correlations, the following theses were formulated: (a) power and velocity measured during the dolphin kick test were strongly or very strongly associated with the height of jumps and lower limb work and power recorded during the jump tests for all swimming distances considered, but none of the jump tests’ parameters showed correlations with work and force during swimming; (b) of all jump tests, the CJ30s test was the one with the strongest correlations and the highest coefficients of determination between jump parameters and the results of the dolphin kick test; (c) the results of the butterfly arms-only test and the butterfly arm pull test were not strongly correlated with each other.

The first finding from our study is a correlation between swimming velocity and power recorded during the dolphin kick test and the height of jumps and lower limb work and power measured by the jump tests, and a lack of correlations between force and work recorded during the swim tests and all three jump parameters. Although jump height is reported to be associated with some elements of competitive swimming, such as starts and turns [38,39], its relationship with swimming with legs only or with both arms and legs has not been confirmed [15]. Our study, which is novel in that it sought correlations between the results of single and continuous jump tests and variables measured during the dolphin kick test, has demonstrated that the height of jumps measured by the CJ15s and CJ30s tests is indeed associated with dolphin kick variables, although less than lower limb work and power. It is important to note here that the first five meters after a push-off were omitted from our analysis to avoid biasing the results of the dolphin kick test. A push-off can be taken as equivalent to a jump off the block, which has been studied by a number of researchers [12,13]. According to our findings, not only lower limb work and power recorded during continuous jumps, but also their height is strongly correlated with swimming variables, the most strongly with swimming velocity. The lack of correlation between force and work measured by the dolphin kick test and the height and lower limb work and power obtained during the jump tests indicates that jump parameters are not useful for monitoring and predicting force and work in the water. Although research points to a relationship between, for instance, work performed during a CMJ and force in legs-only swims [15], our analysis involving as many as 5 jump tests has not found evidence to support its existence.

The best correlations between the results of the jump tests and swim variables were determined in our study for the CJ30s test, meaning that continuous jumps better predict a swimmer’s performance than single jumps. All 36 pairs of correlations between jump height and lower limb power and work and swimming times, velocities and power proved statistically significant for all distances. The best correlations were determined for mean work during the CJ30s test and swimming time and velocity. In this case, *r* values for the first 5 m were 0.90 (*R*^2^ = 0.81). A strong association with this distance was also established for jump height. Čular et al. (2018), who used the CJ30s test to assess the anaerobic power and aerobic capacity in children practising karate, found it to be convenient to apply and yield reliable measurements [32]. The test is deemed the most appropriate for sports based on the stretch shortening cycle (SSC), mainly team and racquet sports. The Wingate Anaerobic Test (WAnT), which is widely used by swimming coaches and researchers, better addresses the needs of cycling, cross-country running, skating, and rowing [40], and its usefulness for swimmers can be questioned on the grounds that the reactions and muscle work it measures are actually different from those associated with swimming [41,42]. The correlations between the results of the CJ30s test and variables measured during the dolphin kick obtained in our study indicate that the CJ30s test can and should be used to seek correlations between the strength of swimmers’ lower limbs and their performance.

Lastly, we did not find correlations between any of the variables measured by the butterfly arms-only test and the results of the butterfly arm pull test. Although the Sprint 1080 is an advanced device that yields reliable measurements of athletes’ biomechanics [43], there is still a degree of uncertainty about its ability to exactly reproduce the butterfly arm pull. Because studies on swimmers using the Sprint 1080 are still few, to ensure maximum reliability of the results, we used a line resistance corresponding to 10% of participants’ body mass based on recognised research reports on resisted running [44,45,46,47]. It is possible that our conclusion that the results of the two tests are not correlated is valid, despite research evidence pointing to strong relationships between swimming with arms only and the results of dynamic arm tests such as those we used [15]. In swimming, strong upper limbs are crucial to generating propulsion and velocity [19]. A plausible explanation for the lack of correlation between results of the butterfly arm pull test and the butterfly arms-only test is that being successful in swimming relies more on technique than strength and power.

The limitations of the study are the small sample consisting of only 12 swimmers and the analysis omitting their preferred swimming strokes. In future research, a larger sample of swimmers should be used to verify these results. Also, given the surprising lack of correlations between the results of the butterfly arm pull test and the variables measured during the swim tests, measurements should be repeated using a device other than the Sprint 1080.

Our study suggests that dry-land training programmes based on carefully selected exercises, such as vertical jumps, may contribute to improvements in competitive swimmers’ performance and provide coaches with one more tool for monitoring their progress. For instance, incorporating exercises that replicate similar movement patterns, or tests such as the CJ30s during land-based training, could be associated with dolphin kick performance, particularly in terms of speed, power, and work efficiency. Decreasing the amount of training time in the pool in favour of an increased proportion of equally effective training on land, the natural environment for humans, may help reduce the incidence of health problems among swimmers and could support their focus on training. Motor skills exercises varying from conventional swimming training may represent an important complement in the future of competitive swimming. Indeed, this appears to be reflected in the steadily improving performance of competitive swimmers, the disappearance of successive barriers, and the establishment of new records.

Although our cohort consisted of junior competitors, the strong dry-land–water links for vertical jumps indicate a potential broader utility. Recreational or fitness-oriented swimmers may adopt simple jump tests to monitor leg-power progress, set concrete goals, and detect early fatigue or asymmetries. Embedding CJ30s or similar protocols in community swim programs could help boost motivation, adherence, and personalized training outcomes. By validating cost-effective, equipment-light methods for early strength assessment and injury prevention, this work may address two public-health priorities: increasing physical activity uptake and reducing sports-related injuries. Simple land-based drills require minimal space and no specialized gear, making them feasible in schools, fitness centers, or rehab clinics. Early identification of power deficits via these tests could prompt timely interventions—targeted resistance work or technique coaching—to mitigate injury risk, promote lifelong activity, and ultimately reduce healthcare burdens.

## 5. Conclusions

Dry-land training programmes, especially those including exercises like vertical jumps or CJ30s tests, can effectively support the improvement of dolphin kick performance in competitive swimmers, particularly in terms of speed, power, and work efficiency. This indicates that coaches may successfully integrate land-based training as a complementary method to traditional water-based sessions.

## Figures and Tables

**Table 1:** Jump height and lower limb work and power obtained during the jump tests.

Jump Test	Height [cm]	Power [W]	Work [J]
SJ	32.78 (6.77)	2829.87 (525.12)	208.22 (46.28)
CMJ	39.28 (6.74)	3224.42 (568.33)	249.96 (51.95)
ACMJ	37.75 (7.81)	3131.85 (578.99)	240.04 (54.04)
CJ15s	27.8 (5.35)	20.03 (3.37)	176.69 (37.08)
CJ30s	25.28 (4.81)	18.6 (3.03)	160.88 (34.55)

**Table 2:** The peak values of power, force and velocity obtained during the butterfly-arm only test.

Arm Tests	Unit
peak power	659.27 (122.97) W
peak force	144.81 (29.25) N
peak velocity	5.05 (1.37) m∙s^−1^

**Table 3:** Time, velocity, power, force and work measured during the dolphin kick test by distance.

Distance [m]	Time [s]	Velocity [m∙s^−1^]	Power [W]	Force [N]	Work [J]
0–5	4.85 (0.56)	1.05 (0.13)	18.52 (2.53)	15.78 (0.62)	88.64 (6.64)
0–10	9.93 (1.16)	1.02 (0.13)	18.05 (2.46)	15.71 (0.59)	176.84 (12.53)
0–15	15.35 (1.80)	0.99 (0.12)	17.52 (2.47)	15.64 (0.59)	264.62 (18.68)
0–20	21.15 (2.53)	0.96 (0.12)	16.98 (2.48)	15.58 (0.60)	352.33 (25.05)

**Table 4:** Pearson’s correlations (*r*) and the coefficients of determination (*R*^2^) between average height of jumps and lower limb power measured by the SJ, CMJ, ACMJ, CJ15s, and CJ30s tests in relation to average time, swimming velocity and power recorded during the 25 m dolphin kick test by distance.

		0–5 m						0–10 m						0–15 m						0–20 m					
		Time [s]		Velocity [m/s]		Power [W]		Time [s]		Velocity [m/s]		Power [W]		Time [s]		Velocity [m/s]		Power [W]		Time [s]		Velocity [m/s]		Power [W]	
JT	VAR	*r*	*R* ^2^	*r*	*R* ^2^	*r*	*R* ^2^	*r*	*R* ^2^	*r*	*R* ^2^	*r*	*R* ^2^	*r*	*R* ^2^	*r*	*R* ^2^	*r*	*R* ^2^	*r*	*R* ^2^	*r*	*R* ^2^	*r*	*R* ^2^
SJ	H [cm]	0.73	0.53	0.79	0.63	0.61	0.38	0.73	0.53	0.79	0.62	0.62	0.38	0.73	0.53	0.79	0.62	0.61	0.38	0.72	0.52	0.78	0.61	0.59	0.35
	P [W]	**0.83**	0.69	**0.87**	0.75	0.66	0.43	**0.84**	0.70	**0.87**	0.76	0.68	0.47	**0.84**	0.71	**0.87**	0.76	0.68	0.46	**0.82**	0.68	**0.86**	0.74	0.66	0.43
	W [J]	**0.82**	0.67	**0.86**	0.74	0.64	0.40	**0.82**	0.67	**0.86**	0.74	0.65	0.43	**0.82**	0.67	**0.86**	0.73	0.65	0.42	**0.81**	0.65	**0.85**	0.72	0.63	0.40
CMJ	H [cm]	0.75	0.56	0.78	0.60	0.60	0.36	0.74	0.55	0.76	0.59	0.60	0.36	0.75	0.56	0.77	0.59	0.60	0.36	0.75	0.57	0.77	0.60	0.61	0.37
	P [W]	0.78	0.60	0.79	0.62	0.60	0.36	0.78	0.61	0.78	0.62	0.61	0.38	0.79	0.62	0.79	0.62	0.61	0.38	0.78	0.61	0.79	0.62	0.62	0.38
	W [J]	0.79	0.62	0.79	0.63	0.58	0.33	0.78	0.61	0.78	0.61	0.59	0.35	0.79	0.62	0.78	0.61	0.59	0.34	0.79	0.62	0.78	0.61	0.59	0.35
ACMJ	H [cm]	0.72	0.52	0.75	0.56	0.54 *	0.29	0.73	0.53	0.75	0.56	0.55 *	0.31	0.71	0.51	0.74	0.55	0.55 *	0.30	0.70	0.50	0.73	0.54	0.53 *	0.28
	P [W]	0.79	0.63	**0.80**	0.64	0.56 *	0.31	**0.80**	0.64	**0.80**	0.65	0.59	0.34	0.79	0.62	**0.80**	0.64	0.59	0.34	0.78	0.60	0.79	0.62	0.56 *	0.32
	W [J]	**0.82**	0.67	**0.83**	0.70	0.60	0.36	**0.84**	0.70	**0.84**	0.71	0.63	0.40	**0.83**	0.69	**0.84**	0.71	0.63	0.40	**0.81**	0.66	**0.83**	0.68	0.61	0.37
CJ15s	H [cm]	0.76	0.58	**0.81**	0.65	0.60	0.36	0.77	0.59	**0.81**	0.66	0.61	0.37	0.75	0.56	**0.80**	0.64	0.60	0.36	0.73	0.53	0.79	0.62	0.58	0.34
	P [W]	**0.82**	0.68	**0.86**	0.74	0.64	0.41	**0.84**	0.70	**0.87**	0.75	0.66	0.43	**0.82**	0.68	**0.86**	0.74	0.66	0.43	**0.81**	0.65	**0.85**	0.72	0.64	0.41
	W [J]	**0.84**	0.71	**0.87**	0.76	0.62	0.39	**0.85**	0.72	**0.87**	0.76	0.64	0.41	**0.84**	0.70	**0.86**	0.74	0.63	0.40	**0.81**	0.66	**0.85**	0.72	0.62	0.38
CJ30s	H [cm]	**0.85**	0.72	**0.87**	0.76	0.64	0.41	**0.84**	0.71	**0.87**	0.75	0.64	0.41	**0.83**	0.70	**0.86**	0.74	0.64	0.41	**0.83**	0.68	**0.85**	0.73	0.63	0.40
	P [W]	**0.87**	0.76	**0.88**	0.78	0.64	0.41	**0.88**	0.77	**0.88**	0.78	0.66	0.43	**0.87**	0.76	**0.88**	0.78	0.66	0.44	**0.87**	0.75	**0.88**	0.77	0.65	0.43
	W [J]	**0.90**	**0.80**	**0.90**	0.81	0.63	0.40	**0.89**	0.79	**0.89**	**0.80**	0.65	0.42	**0.88**	0.78	**0.89**	0.79	0.65	0.42	**0.87**	0.76	**0.88**	0.78	0.64	0.41

Note: JT—jump test; VAR—variable; SJ—squat jump; CMJ—countermovement jump; ACMJ—akimbo countermovement jump; CJ15s—15 s of continuous jumps; CJ30s—30 s of continuous jumps; values with an asterisk indicate non-significant correlations (*p* > 0.05); and bold indicates strong correlations (*r* ≥ 0.80).

## Data Availability

The datasets generated during this investigation are available from the corresponding author on reasonable request. The raw data supporting the conclusions of this article will be made available by the authors on request.
